# Prevalence and Pattern of Thyroid Dysfunction Among Women of Reproductive Age With Abnormal Uterine Bleeding: A Prospective Observational Study

**DOI:** 10.7759/cureus.116209

**Published:** 2026-09-14

**Authors:** Harshita Mishra, Richa Rathoria, Rekha Devi, Ekta Chaudhary, Mahboob Ahmad, Vishi Rawat, Ekansh Rathoria, Kusum Lata

**Affiliations:** 1 Obstetrics and Gynecology, Hind Institute of Medical Sciences, Sitapur, IND; 2 Biochemistry, Hind Institute of Medical Sciences, Sitapur, IND; 3 Pediatrics, Hind Institute of Medical Sciences, Sitapur, IND

**Keywords:** abnormal uterine bleeding, hyperthyroidism, hypothyroidism, menorrhagia, premenopausal women, thyroid diseases

## Abstract

Introduction

Abnormal uterine bleeding (AUB) is a common gynecological condition that adversely affects the quality of life of women of reproductive age. Thyroid dysfunction is a recognized but often underdiagnosed non-structural endocrine cause of AUB that may be amenable to medical treatment. This study aimed to determine the prevalence and pattern of thyroid dysfunction among reproductive-aged women with AUB and evaluate its association with demographic characteristics, body mass index (BMI), thyroid hormone profile, and International Federation of Gynaecology and Obstetrics (FIGO) menstrual bleeding patterns.

Methodology

This prospective observational study was conducted among 150 reproductive-aged women (18-45 years) presenting with AUB at a tertiary care hospital in North India. Consecutive sampling was used for participant enrolment. Women with structural causes of AUB were excluded after clinical evaluation and ultrasonography according to the FIGO PALM-COEIN classification. Thyroid function was assessed using serum thyroid-stimulating hormone (TSH), total triiodothyronine (T3), and total thyroxine (T4). Participants were categorized into euthyroid and thyroid dysfunction groups, and clinicodemographic characteristics, BMI, thyroid hormone profiles, and menstrual bleeding patterns were compared. Multivariable binary logistic regression was performed to identify factors independently associated with thyroid dysfunction and menorrhagia.

Results

Thyroid dysfunction was identified in 45 of 150 women (30.0%). Overt hypothyroidism was the most common subtype (19/45, 42.2%), followed by subclinical hypothyroidism (16/45, 35.6%) and overt hyperthyroidism (10/45, 22.2%). No cases of subclinical hyperthyroidism were identified. Thyroid dysfunction was significantly associated with age >30 years (*p* = 0.044) and BMI ≥25 kg/m² (*p* < 0.001). On multivariable analysis, BMI ≥25 kg/m² remained independently associated with thyroid dysfunction (aOR, 33.33; 95% CI, 12.35-90.91; *p* < 0.001), whereas age >30 years was not (aOR, 2.18; 95% CI, 0.79-6.02; *p* = 0.135). Women with thyroid dysfunction had significantly higher mean serum TSH levels than euthyroid women (6.20 ± 4.89 vs. 2.54 ± 0.96 mIU/L; *p* < 0.001), whereas T3 and T4 levels were comparable. Significant associations were observed between thyroid dysfunction subtypes and FIGO menstrual bleeding patterns (*p* < 0.001), with menorrhagia predominating in overt hypothyroidism and polymenorrhagia in subclinical hypothyroidism and overt hyperthyroidism. On multivariable analysis adjusted for age and BMI, women with thyroid dysfunction were significantly less likely to present with menorrhagia compared to euthyroid controls (aOR, 0.06; 95% CI, 0.02-0.20; *p* < 0.001).

Conclusion

Thyroid dysfunction was common among women of reproductive age presenting with AUB and was significantly associated with BMI ≥25 kg/m² and distinct menstrual bleeding patterns, while the association with age was not sustained after adjustment. Thyroid function testing, particularly serum TSH estimation, should be considered in the evaluation of selected women with AUB based on clinical suspicion and guideline-based evaluation, rather than universal screening, to facilitate early diagnosis and appropriate management of reversible endocrine causes and support individualized treatment decisions.

## Introduction

Abnormal uterine bleeding (AUB) is a prevalent gynaecological disorder affecting up to 30% of reproductive-aged women globally [1,2]. Defined by the International Federation of Gynaecology and Obstetrics (FIGO) as any deviation from normal menstrual frequency, duration, regularity, or blood volume, AUB causes severe chronic morbidity, iron-deficiency anemia, and diminished quality of life [2,3]. While structural causes under FIGO's PALM categories are routinely evaluated using imaging, non-structural endocrine causes, particularly thyroid dysfunction, may remain undiagnosed in women without overt clinical symptoms, leading to delayed or inappropriate clinical interventions [1,4].

Thyroid hormones, i.e., triiodothyronine (T3) and thyroxine (T4), act as crucial regulators of female reproductive physiology [4]. They modulate gonadotropin-releasing hormone (GnRH) pulsatility, hepatic sex hormone-binding globulin (SHBG) synthesis, and peripheral estrogen metabolism [4,5]. Systematic reviews demonstrate that both overt and subclinical thyroid disorders disrupt hypothalamic-pituitary-ovarian axis signalling, precipitating anovulatory cycles, progesterone insufficiency, and unsynchronized endometrial shedding [1,6-8]. Subclinical hypothyroidism often lacks classical systemic signs yet silently alters menstrual bleeding patterns [5,7]. Because thyroid dysfunction often remains clinically unrecognized in women of reproductive age, establishing its contribution to AUB is of considerable clinical importance [3,8].

Screening serum thyroid parameters (T3, T4, thyroid-stimulating hormone (TSH)) provides an accessible, non-invasive, and cost-effective diagnostic step [9,10]. Early detection of thyroid dysfunction enables targeted medical correction, restoring normal ovulatory cycles and resolving abnormal bleeding without necessitating invasive surgical procedures or empirical hormonal therapy [10-12]. Routine assessment of thyroid function may facilitate identification of an underlying endocrine cause in selected women presenting with AUB and may reduce unnecessary hormonal therapy or surgical interventions [10,12].

While the association between thyroid disease and menstrual disturbance is broadly recognized, prospective regional data evaluating the association between thyroid dysfunction and specific menstrual bleeding patterns across distinct thyroid subtypes (overt vs. subclinical hypothyroidism vs. hyperthyroidism) in North Indian populations remain limited [6,9,13]. Although the association between thyroid dysfunction and menstrual abnormalities has been reported in several Indian hospital-based studies, these studies were largely limited by relatively small sample sizes or single-center designs and provided limited characterization of menstrual bleeding patterns across different thyroid dysfunction subtypes [14,15].

Furthermore, prospective data from North India remain scarce. Therefore, the present study was undertaken with the primary objective of determining the prevalence of overt and subclinical thyroid dysfunction among reproductive-aged women presenting with AUB in a tertiary care hospital in North India. The secondary objectives were to evaluate the association between thyroid status and menstrual bleeding patterns.

## Materials and methods

Study design and setting

This prospective observational study was conducted in the Department of Obstetrics and Gynaecology, in collaboration with the Department of Biochemistry, at Hind Institute of Medical Sciences, Sitapur, Uttar Pradesh, India, over a period of 18 months from August 2024 to January 2026.

Ethical approval

The study was approved by the Institutional Ethics Committee of Hind Institute of Medical Sciences, Sitapur, before participant enrolment (Approval No.: IHEC-HIMSA/MD/MS-23/RD-21/07-24 dated 24/07/2024). Written informed consent was obtained from all participants before inclusion in the study.

Study population

Women aged 18-45 years presenting with AUB to the outpatient or inpatient Department of Obstetrics and Gynaecology during the study period were screened for eligibility.

Inclusion criteria

Women aged 18-45 years presenting with AUB, as defined by FIGO, and who consented to participate were included in the study [9].

Exclusion criteria

Women who were pregnant or in the postpartum period were excluded from the study. Patients with known structural causes of AUB, including uterine fibroids, adenomyosis, endometrial polyps, or genital tract malignancy, were also excluded. Additional exclusion criteria included a previous diagnosis of thyroid disease, current treatment with thyroid hormone replacement or antithyroid medications, use of hormonal therapy, anticoagulants, or other medications known to affect menstrual bleeding or thyroid function, and the presence of bleeding disorders, chronic liver disease, chronic kidney disease, or other systemic illnesses associated with menstrual abnormalities.

Sample size and sampling

Based on a previously reported prevalence of thyroid dysfunction of 10.66% by Subedi et al., with a 95% confidence level, 5% absolute precision, and a design effect of 1, the calculated minimum sample size was 147 using OpenEpi software [16,17]. A total of 150 participants were ultimately included in the study. All eligible women were enrolled during the study period using a consecutive sampling technique.

Data collection

A detailed clinical history was obtained from each participant using a predesigned, structured proforma. Demographic and clinical data, including age, parity, socioeconomic status, obstetric history, contraceptive use, duration and pattern of AUB, and relevant medical history, were recorded. For statistical analysis, age was categorized as ≤30 years and >30 years to compare thyroid dysfunction across younger and relatively older reproductive-aged women. Anthropometric measurements, including weight (kg) and height (m), were obtained. BMI was calculated as weight in kilograms divided by height in meters squared (kg/m²) and categorized as <25 kg/m² and ≥25 kg/m² for statistical analysis, corresponding to the conventional WHO threshold for overweight in adults [18]. All participants underwent a comprehensive general physical, systemic, and gynecological examination. Menstrual bleeding patterns were classified according to the FIGO PALM-COEIN classification system [9]. Pelvic ultrasonography, either transvaginal or transabdominal as appropriate, was performed to evaluate the uterus and adnexa and to exclude structural causes (PALM category) of AUB [10].

Laboratory investigations and diagnostic definitions

Fasting venous blood samples (5 mL) were collected from all participants under aseptic precautions for the estimation of serum TSH, T3, and T4 using a chemiluminescent immunoassay (CLIA). Thyroid function was assessed using serum TSH, total T3, and total T4. Free T3 (FT3) and free T4 (FT4) were not assessed because these assays were not available at the study facility. 

Thyroid function was interpreted according to the hospital laboratory reference ranges (total T3: 80-200 nanograms per deciliter (ng/dL), total T4: 5-12 micrograms per deciliter (µg/dL), and TSH: 0.4-4.0 milli-international units per liter (mIU/L)), which were consistent with established adult laboratory reference ranges [19]. Thyroid dysfunction was therefore classified using serum TSH, total T3, and total T4 according to predefined biochemical criteria [11].

Euthyroidism was defined as serum TSH, T3, and T4 levels within the hospital laboratory reference ranges [11]. Subclinical hypothyroidism was defined as elevated TSH with T3 and T4 levels within the reference ranges, whereas overt hypothyroidism was defined as elevated TSH with reduced T4 levels [11]. Overt hyperthyroidism was defined as suppressed TSH with elevated T3 and/or T4 levels, whereas subclinical hyperthyroidism was defined as suppressed TSH with T3 and T4 levels within the reference ranges [11].

Statistical analysis

Data were entered into Microsoft Excel (Microsoft Corporation, Redmond, WA, US) and analyzed using IBM SPSS Statistics version 26.0 (IBM Corp., Armonk, NY, USA). Categorical variables were expressed as frequencies and percentages, while continuous variables were presented as mean ± standard deviation (SD). Associations between categorical variables were assessed using the Chi-square test or Fisher’s exact test, as appropriate. For contingency tables with more than two categories and sparse cell frequencies, the Fisher-Freeman-Halton exact test was used. Continuous variables between two independent groups were compared using Welch’s independent-samples t-test.

Two multivariable binary logistic regression models were performed using the Enter method. The first model evaluated factors independently associated with thyroid dysfunction, with age group and BMI category entered as predictors (reference categories: age ≤30 years and BMI <25 kg/m²). The second model evaluated factors independently associated with menorrhagia, with age group, BMI category, and thyroid status entered as predictors (reference categories: age ≤30 years, BMI <25 kg/m2, and euthyroid). Adjusted odds ratios (aORs) with 95% confidence intervals (CIs) were reported. Model fit was assessed using the omnibus test of model coefficients, −2 log likelihood, Cox and Snell R², and Nagelkerke R². All statistical tests were two-tailed, and a p-value <0.05 was considered statistically significant.

## Results

A total of 150 women with AUB were enrolled in the study. Of these, 105/150 (70.0%) were euthyroid and 45/150 (30.0%) had thyroid dysfunction. Women with thyroid dysfunction were significantly more likely to be older than 30 years than euthyroid women (40.0% (18/45) vs. 23.8% (25/105), p=0.044). No significant associations were observed with family history of thyroid disease, marital status, parity, number of living children, or history of abortion (all p>0.05) (Table 1).

**Table 1:** Baseline demographic and obstetric characteristics of women with abnormal uterine bleeding according to thyroid status (n=150) Data are presented as n (%); χ² = Chi-square test; Significant p value <0.05

Variable	Category	Euthyroid (n = 105)	Thyroid dysfunction (n = 45)	χ² value	P value
Age group (years)	≤30	80 (76.2)	27 (60.0)	4.038	0.044
	>30	25 (23.8)	18 (40.0)		
Family history of thyroid disease	No	88 (83.8)	39 (86.7)	0.198	0.656
	Yes	17 (16.2)	6 (13.3)		
Marital status	Married	84 (80.0)	38 (84.4)	0.410	0.522
	Unmarried	21 (20.0)	7 (15.6)		
Parity	Nulliparous	24 (22.9)	10 (22.2)	0.646	0.886
	One	19 (18.1)	6 (13.3)		
	Two–Three	39 (37.1)	19 (42.2)		
	> Three	23 (21.9)	10 (22.2)		
Living children	None	25 (23.8)	11 (24.4)	0.868	0.833
	One	23 (21.9)	7 (15.6)		
	Two–Three	41 (39.0)	20 (44.4)		
	> Three	16 (15.2)	7 (15.6)		
History of abortion	None	85 (81.0)	33 (73.3)	3.068	0.381
	One	17 (16.2)	9 (20.0)		
	Two	2 (1.9)	3 (6.7)		
	Three	1 (1.0)	0 (0.0)		

Women with thyroid dysfunction had a significantly higher prevalence of overweight or obesity (BMI ≥25 kg/m²) than euthyroid women (73.3% (33/45) vs. 7.6% (8/105), p < 0.001). Menorrhagia was the predominant bleeding pattern among euthyroid women, whereas polymenorrhagia, hypomenorrhea, and oligomenorrhea were more frequently observed in women with thyroid dysfunction (p<0.001). Age at menarche and duration of symptoms were comparable between the groups (both p>0.05) (Table 2).

**Table 2:** Clinical characteristics and menstrual bleeding patterns of women with abnormal uterine bleeding according to thyroid status (n=150) Data are presented as n (%); χ² = Chi-square test; Significant p value <0.05

Parameter	Category	Euthyroid (n = 105)	Thyroid dysfunction (n = 45)	χ² value	P value
Body mass index (kg/m²)	<25	97 (92.4)	12 (26.7)	68.486	<0.001
	≥25	8 (7.6)	33 (73.3)		
Age at menarche (years)	10	0 (0.0)	1 (2.2)	3.221	0.522
	11	5 (4.8)	1 (2.2)		
	12	39 (37.1)	15 (33.3)		
	13	41 (39.0)	20 (44.4)		
	14	20 (19.0)	8 (17.8)		
Pattern of abnormal uterine bleeding	Menorrhagia	93 (88.6)	18 (40.0)	48.430	<0.001
	Metrorrhagia	1 (1.0)	3 (6.7)		
	Polymenorrhagia	3 (2.9)	16 (35.6)		
	Hypomenorrhea	0 (0.0)	3 (6.7)		
	Oligomenorrhea	5 (4.8)	4 (8.9)		
	Menometrorrhagia	3 (2.9)	1 (2.2)		
Duration of symptoms (years)	<5	76 (72.4)	29 (64.4)	0.945	0.331
	≥5	29 (27.6)	16 (35.6)		

Comparison of continuous clinical characteristics showed that women with thyroid dysfunction were significantly older than euthyroid women (28.98 ± 5.93 vs. 26.95 ± 5.21 years; p=0.038). They also had a significantly higher mean body mass index (24.89 ± 4.37 vs. 22.29 ± 2.30 kg/m²; p<0.001). In contrast, the mean age at menarche and the mean duration of symptoms were comparable between the two groups, with no statistically significant differences (Table 3).

**Table 3:** Comparison of continuous clinical characteristics between euthyroid women and women with thyroid dysfunction (n=150) Data are presented as mean ± standard deviation (SD); Comparisons between groups were performed using Welch’s independent samples t-test; significant p-value <0.05.

Parameter	Euthyroid (n = 105)	Thyroid dysfunction (n = 45)	t value	P value
Age (years), Mean ± SD	26.95 ± 5.21	28.98 ± 5.93	2.091	0.038
Body mass index (kg/m²), Mean ± SD	22.29 ± 2.30	24.89 ± 4.37	4.757	<0.001
Age at menarche (years), Mean ± SD	12.72 ± 0.83	12.73 ± 0.86	0.064	0.949
Duration of symptoms (years), Mean ± SD	4.22 ± 1.05	4.24 ± 1.42	0.121	0.904

Serum TSH levels were significantly higher among women with thyroid dysfunction than euthyroid controls (6.20 ± 4.89 vs. 2.54 ± 0.96 mIU/L; p < 0.001), whereas mean T3 (158.87 ± 102.18 vs. 153.15 ± 29.43 ng/dL; p = 0.714) and T4 levels (8.24 ± 3.70 vs. 7.68 ± 1.71 µg/dL; p = 0.334) did not differ significantly between the groups (Table 4).

**Table 4:** Comparison of thyroid hormone profile between euthyroid women and women with thyroid dysfunction (n=150) Data are presented as mean ± standard deviation (SD). Groups were compared using Welch’s t-test. Reference ranges are based on the hospital laboratory reference ranges. p < 0.05 was considered statistically significant.

Parameter	Reference range	Euthyroid (n=105) Mean ± SD	Thyroid dysfunction (n=45) Mean ± SD	t value	p-value
TSH (mIU/L)	0.4–4.0	2.54 ± 0.96	6.20 ± 4.89	−4.983	<0.001
T3 (ng/dL)	80–200	153.15 ± 29.43	158.87 ± 102.18	−0.369	0.714
T4 (µg/dL)	5–12	7.68 ± 1.71	8.24 ± 3.70	−0.975	0.334

Among the 45 women with thyroid dysfunction, overt hypothyroidism was the predominant subtype (19/45, 42.2%), followed by subclinical hypothyroidism (16/45, 35.6%) and overt hyperthyroidism (10/45, 22.2%) (Figure 1). No participant had subclinical hyperthyroidism.

**Figure 1:**
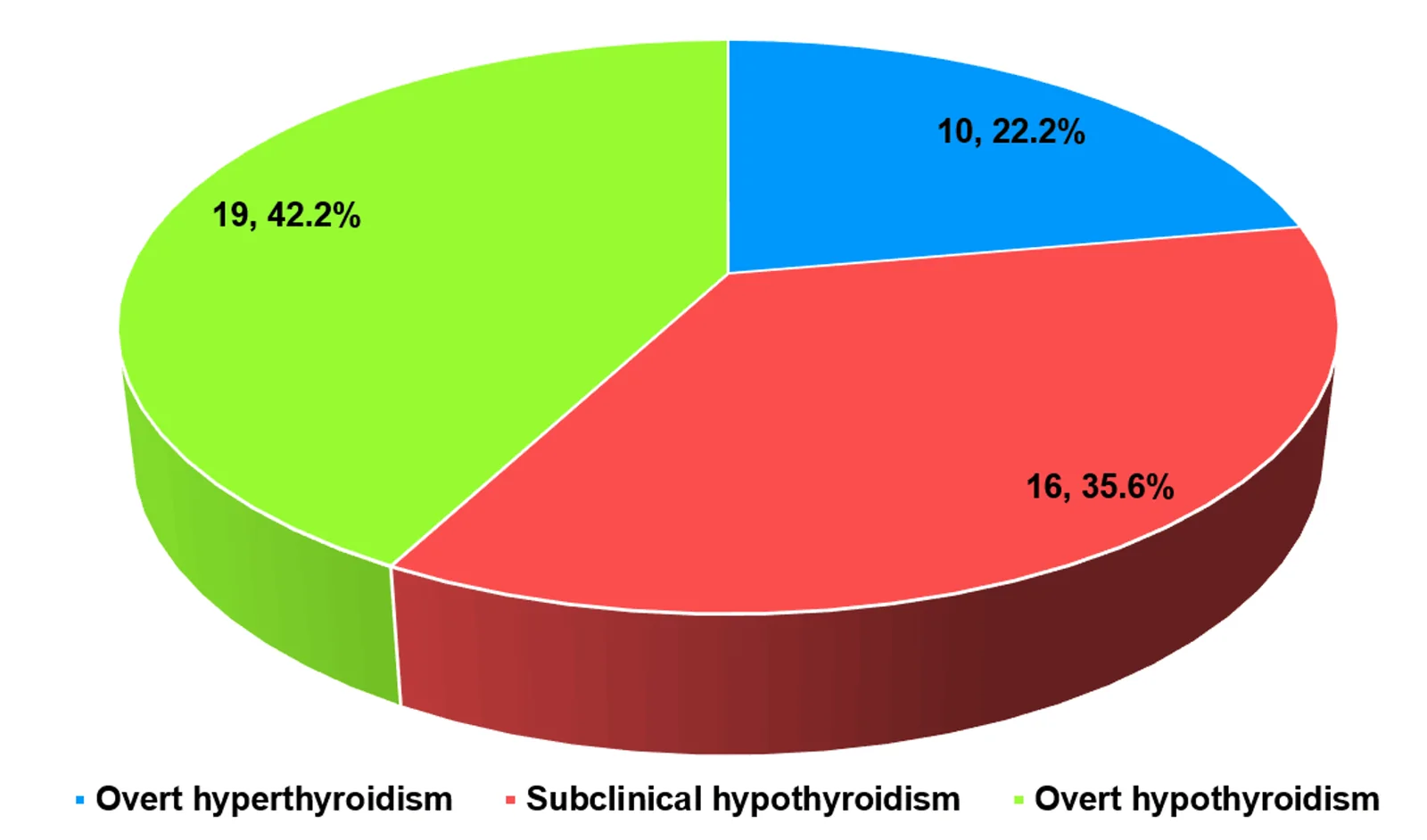
Distribution of thyroid dysfunction among women with abnormal uterine bleeding (n = 45)

Among women with thyroid dysfunction, menstrual bleeding patterns differed significantly according to thyroid dysfunction subtype (p<0.001). Menorrhagia predominated in women with overt hypothyroidism (15/19, 78.9%), whereas polymenorrhagia was the most frequent presentation in women with overt hyperthyroidism (6/10, 60.0%) and subclinical hypothyroidism (8/16, 50.0%). Hypomenorrhea occurred exclusively in women with overt hyperthyroidism, while oligomenorrhea was observed more frequently in subclinical hypothyroidism (Table 5). No cases of subclinical hyperthyroidism were identified.

**Table 5:** Association between thyroid dysfunction subtype and menstrual bleeding pattern among women with abnormal uterine bleeding (n = 45) Data are presented as n (%); the association between thyroid dysfunction subtype and menstrual bleeding pattern was assessed using the Fisher-Freeman-Halton exact test; significant p-value <0.05. No cases of subclinical hyperthyroidism were identified; therefore, this category was not included in the subtype-specific analysis.

Bleeding pattern	Overt hyperthyroidism (n = 10)	Subclinical hypothyroidism (n = 16)	Overt hypothyroidism (n = 19)	Statistical test	P value
Menorrhagia	0 (0.0)	3 (18.8)	15 (78.9)	Fisher–Freeman–Halton exact test	<0.001
Metrorrhagia	0 (0.0)	2 (12.5)	1 (5.3)		
Polymenorrhagia	6 (60.0)	8 (50.0)	2 (10.5)		
Hypomenorrhea	3 (30.0)	0 (0.0)	0 (0.0)		
Oligomenorrhea	1 (10.0)	3 (18.8)	0 (0.0)		
Menometrorrhagia	0 (0.0)	0 (0.0)	1 (5.3)		

On multivariable logistic regression, BMI ≥25 kg/m² remained independently associated with thyroid dysfunction (adjusted OR (aOR), 33.33; 95% CI, 12.35-90.91; p<0.001). Age >30 years was not independently associated with thyroid dysfunction (aOR, 2.18; 95% CI, 0.79-6.02; p=0.135) (Table 6).

**Table 6:** Multivariable logistic regression analysis of factors associated with thyroid dysfunction (n=150) OR, odds ratio; CI, confidence interval; Dependent variable: thyroid dysfunction (1 = thyroid dysfunction, 0 = euthyroid); Reference categories: age ≤30 years and BMI <25 kg/m²; Model adjusted for age group and BMI category; Omnibus model χ² = 69.433, df = 2, p < 0.001; −2 log likelihood = 113.826; Cox and Snell R² = 0.371; Nagelkerke R² = 0.525.

Predictor	Adjusted OR	95% CI	p-value
Age >30 years	2.18	0.79–6.02	0.135
BMI ≥25 kg/m²	33.33	12.35–90.91	<0.001

On multivariable logistic regression, thyroid dysfunction was independently associated with a significantly lower likelihood of presenting with menorrhagia compared to euthyroid controls (aOR, 0.06; 95% CI, 0.02- 0.20; p<0.001). Age >30 years and BMI ≥25 kg/m² were not independently associated with menorrhagia (Table 7).

**Table 7:** Multivariable logistic regression analysis of factors associated with menorrhagia (n=150) OR, odds ratio; CI, confidence interval; Dependent variable: menorrhagia (1=menorrhagia, 0=other AUB patterns); Reference categories: age ≤30 years, BMI<25 kg/m2, and euthyroid; Model adjusted for age, BMI, and thyroid status. Model fit statistics: Omnibus χ2=38.242 (df=3,p<0.001); -2 Log likelihood=133.675; Cox & Snell R2=0.225; Nagelkerke R2=0.330.

Predictor	Category / Comparison	Adjusted OR	95% CI	p-value
Age Group	>30 years vs. <=30 years	1.36	0.55–3.38	0.505
BMI Category	>=25 Kg/m^2 ^vs. <25 Kg/m^2^	0.51	0.14–1.84	0.305
Thyroid Status	Thyroid Dysfunction vs. Euthyroid	0.06	0.02 – 0.20	<0.001

## Discussion

In the present study, thyroid dysfunction was identified in 30.0% (45/150) of reproductive-aged women presenting with AUB. This prevalence is comparable to that reported by Sangeetha et al. (28.5%) and is consistent with the findings of Sahu et al., who highlighted the importance of non-structural endocrine causes in women presenting with AUB [5,8]. Similar associations between thyroid dysfunction and menstrual abnormalities have been reported by Chowdhury et al., Yatish et al., Kolli et al., Bedi et al., and Sureka et al. [12-15,20]. In contrast, higher prevalence rates were reported by Kitahara et al., MacGregor et al., and Tan et al. [9-11]. These differences may reflect variations in study populations, referral patterns, iodine sufficiency, and diagnostic criteria.

Thyroid dysfunction was significantly more frequent among women aged >30 years on univariate analysis (p = 0.044) (Table 1), consistent with the findings of Himabindu et al., who reported a higher frequency of overt and subclinical hypothyroidism among women aged 31-40 years with menstrual irregularities [7]. However, age >30 years was not independently associated with thyroid dysfunction after adjustment for BMI (aOR, 2.18; 95% CI, 0.79-6.02; p = 0.135). Parity, marital status, and history of abortion were also not significantly associated with thyroid dysfunction (p > 0.05), consistent with the findings of Kolli et al. [14].

Women with thyroid dysfunction had a significantly higher mean BMI than euthyroid women (24.89 ± 4.37 vs. 22.29 ± 2.30 kg/m²; p < 0.001), and BMI ≥25 kg/m² was significantly associated with thyroid dysfunction (Tables 2, 3). This finding is consistent with the recognized bidirectional relationship between thyroid dysfunction and obesity, whereby hypothyroidism may predispose to weight gain, while excess adiposity may influence thyroid hormone metabolism and TSH regulation [1,21]. Kulkarni et al. similarly highlighted the interplay between adiposity, insulin resistance, and thyroid dysfunction in women with reproductive disorders [1]. Importantly, BMI ≥25 kg/m² remained independently associated with thyroid dysfunction after adjustment for age (aOR, 33.33; 95% CI, 12.35-90.91; p < 0.001), although the wide confidence interval warrants cautious interpretation of the magnitude of this association. Nevertheless, the observational design precludes determining the directionality of this relationship.

Serum TSH levels were significantly higher among women with thyroid dysfunction than euthyroid women (6.20 ± 4.89 vs. 2.54 ± 0.96 mIU/L; p < 0.001), whereas mean T3 and T4 levels did not differ significantly between the groups (Table 4). Similar findings have been reported by MacGregor et al. and Tan et al., with TSH serving as an important biochemical marker of thyroid dysfunction, particularly in subclinical disease, where T3 and T4 may remain within the reference range [10,11]. These findings support consideration of serum TSH as an important initial biochemical test when thyroid dysfunction is clinically suspected in women presenting with AUB [22].

Overt hypothyroidism was the predominant thyroid dysfunction subtype (19/45, 42.2%), followed by subclinical hypothyroidism (16/45, 35.6%) and overt hyperthyroidism (10/45, 22.2%); no cases of subclinical hyperthyroidism were identified (Figure 1). This distribution is consistent with the findings of Asghar et al. and Tan et al., who also reported hypothyroidism as the most common thyroid abnormality among women with menstrual disorders [6,11].

A significant association was observed between thyroid dysfunction subtypes and FIGO menstrual bleeding patterns (p <0.001) (Table 5). Menorrhagia predominated in overt hypothyroidism (15/19, 78.9%), whereas polymenorrhagia was the most frequent presentation in subclinical hypothyroidism (8/16, 50.0%) and overt hyperthyroidism (6/10, 60.0%). Hypomenorrhea occurred exclusively in women with overt hyperthyroidism, while oligomenorrhea was observed predominantly in subclinical hypothyroidism. These differences may reflect the effects of thyroid hormones on ovulation, sex hormone metabolism, and endometrial function [4,6,12,23]. Similar associations between thyroid dysfunction and specific menstrual abnormalities have been reported by Chowdhury et al. and Begum et al. [12,24].

In multivariable analysis, thyroid dysfunction was independently associated with a significantly lower likelihood of presenting with menorrhagia compared to euthyroid women after adjustment for age and BMI (aOR, 0.06; 95% CI, 0.02-0.20; p <0.001). This reflects the broader clinical spectrum of bleeding abnormalities observed among women with thyroid dysfunction, who presented more frequently with polymenorrhagia, hypomenorrhea, and oligomenorrhea rather than isolated menorrhagia (Table 5). Conversely, euthyroid women in this cohort presented predominantly with menorrhagia (93/105, 88.6%, Table 2). Age and BMI were not independently associated with menorrhagia in the regression model (Table 7).

Structural causes of AUB should be excluded using appropriate diagnostic modalities, including endometrial biopsy when clinically indicated, while coexisting cervical pathology should also be considered with appropriate cervical cancer screening [25,26]. Evaluation should also consider non-structural endocrine causes such as thyroid dysfunction.

These findings have relevance to women's health priorities in India, where heavy menstrual bleeding contributes to iron-deficiency anemia, an important public health concern addressed through the *Anemia Mukt Bharat* (AMB or *Anemia Free India*) strategy of the Ministry of Health and Family Welfare, Government of India [27]. Identification of reversible endocrine causes of AUB may therefore contribute to timely management and reduction of chronic menstrual blood loss.

Furthermore, our findings support consideration of thyroid function testing in selected women with AUB. This approach aligns with the broader objectives of India's National Programme for Prevention and Control of Non-Communicable Diseases (NP-NCD), which emphasizes the early detection and management of non-communicable diseases through strengthened healthcare services [28]. Although the NP-NCD does not currently recommend universal thyroid screening for women with AUB, thyroid function testing in clinically selected women may facilitate early identification and appropriate management of overt and subclinical thyroid dysfunction [3,10].

Strengths

The strengths of this study include its prospective design, standardized assessment of AUB using the FIGO PALM-COEIN classification, systematic exclusion of structural causes by ultrasonography, and detailed clinicobiochemical correlation of thyroid dysfunction subtypes with menstrual bleeding patterns. Biochemical assessment of thyroid function in all participants also strengthened diagnostic consistency.

Limitations

This study has several limitations. First, it was a single-center study conducted at a tertiary care hospital in North India with a relatively modest sample size, which may limit generalizability. Second, FT3 and FT4 were not assessed because these assays were unavailable at the study facility; thyroid dysfunction was therefore classified using serum TSH, total T3, and total T4. While serum TSH remains the primary and most sensitive biomarker for detecting subclinical thyroid disorders, total thyroid hormone levels can be influenced by alterations in thyroid-binding proteins (such as SHBG or thyroxine-binding globulin), making the lack of free hormone estimates (FT3/FT4) a potential limitation that could lead to subtle misclassification or under-characterization of subclinical thyroid status in individual patients. Third, the observational design precludes establishing a causal relationship between thyroid dysfunction and menstrual bleeding patterns. Multivariable models were adjusted for selected covariates (age and BMI); therefore, residual confounding cannot be completely excluded. Fourth, while the sample size of 150 was statistically adequate to estimate the overall prevalence of thyroid dysfunction, the resulting numbers within specific thyroid dysfunction subtypes (e.g., overt hyperthyroidism) and rare bleeding patterns were relatively small; therefore, subtype-specific comparisons should be interpreted with caution as the study may be underpowered for these secondary subgroup analyses. Additionally, the relatively small number of women with BMI ≥25 kg/m² and the wide confidence interval around its adjusted odds ratio warrant cautious interpretation of the magnitude of this association. Finally, the absence of long-term follow-up after treatment precluded an assessment of subsequent menstrual outcomes and the time required for normalization of menstrual cycles after correction of thyroid dysfunction.

## Conclusions

This study demonstrated a high prevalence (30.0%) of thyroid dysfunction among reproductive-aged women presenting with AUB, with overt and subclinical hypothyroidism accounting for most endocrine abnormalities. Thyroid dysfunction was significantly associated with elevated BMI (≥25 kg/m²) and distinct FIGO menstrual bleeding patterns. Menorrhagia predominated among euthyroid women and those with overt hypothyroidism, and polymenorrhagia predominated in subclinical hypothyroidism and overt hyperthyroidism. Although age >30 years was associated with thyroid dysfunction on unadjusted analysis, it was not independently associated after adjustment for BMI. Serum TSH showed the most prominent biochemical difference between women with thyroid dysfunction and euthyroid women in our study. These findings support consideration of thyroid function testing in selected women with AUB based on clinical suspicion and guideline-based evaluation, rather than universal screening, to facilitate early diagnosis and appropriate medical management of reversible endocrine causes.
